# Are clinical outcomes affected by type of plate used for management of mid-shaft clavicle fractures?

**DOI:** 10.1186/s10195-018-0492-0

**Published:** 2018-08-15

**Authors:** Mohammad M. Alzahrani, Adam Cota, Khalid Alkhelaifi, Aljarrah Aleidan, Gregory Berry, Rudy Reindl, Edward Harvey

**Affiliations:** 1 Division of Orthopaedic Surgery, McGill University, 1650 Cedar Ave, Montreal, QC H3G 1A4 Canada; 2 Department of Orthopaedic Surgery, Imam Abdulrahman Bin Faisal University, Dammam, Saudi Arabia; 3 Division of Orthopaedic Trauma, Department of Orthopaedic Surgery, Al-Razi Orthopaedic Hospital, Kuwait City, Kuwait; 4 Rocky Mountain Orthopaedic Associates, Grand Junction, CO USA; 5 Department of Orthopedic Surgery, ASPETAR, Orthopaedic and Sports Medicine Hospital, Doha, Qatar

**Keywords:** Clavicle, Mid-shaft, Fracture, Open reduction internal fixation, Complications, Re-operation, Union

## Abstract

**Background:**

Open reduction and internal fixation (ORIF) using plate osteosynthesis for midshaft clavicle fractures is often complicated by the prominence of the implant due to the subcutaneous position of the clavicle. Reoperation rates for symptomatic clavicle plate removal have been reported to be as high as 53%. We sought to determine to which degree do clinical outcomes (all cause reoperation rate and rate of fracture union) differ between types of clavicle plates.

**Materials and methods:**

A retrospective chart review was performed using our hospital database for patients treated with ORIF for mid-shaft clavicle fractures (OTA/AO type 15-B). Implants included in this review were 2.7 mm reconstruction plates, 3.5 mm reconstruction plates, 3.5 mm precontoured clavicle plates and 3.5 mm locking compression plates. The primary outcome measure was the all cause reoperation rate. Secondary outcomes compared the rate fracture union, documented infection, hardware failures and clinical symptoms at the surgical site among the various plate types. Data was collected and descriptive statistics were analyzed. *p* values < 0.05 were considered statistically significant.

**Results:**

A total of 102 midshaft clavicle fractures treated with ORIF were included in this study. The majority of patients were ≤ 50 years old (83.3%) and male (72.5%). The overall union rate for all plating constructs was 97.1%. We found that age, sex and smoking were not associated with the rate of re-operation. In addition, the fracture classification, type of implant used and number of screws used didn’t increase the risk of revision surgery. In addition, more than 50% of patients complaining of pain at 6 weeks post-operatively required a second surgery for removal of hardware. Moreover, there was no association between age, sex, smoking, fracture classification or plate type and the rate of union. Interestingly, clavicle fractures fixed with 3.5 mm reconstruction plates were more likely to have hardware failure due to plastic deformation, whereas 2.7 mm plates were more likely to fail by plate breakage.

**Conclusion:**

Although different types of implants have different biomechanical properties, no difference in reoperation, union and plate removal rates were found between the various plate types. Future studies with a larger sample size are required to further examine these outcomes.

**Level of evidence:**

Level III.

## Introduction

Middle third clavicle fractures account for up to 80% of all clavicle fractures [1–3]. Although historically the majority of these injuries have been treated non-operatively, surgical fixation has been shown to improve clinical outcomes, especially in displaced fracture patterns with significant shortening [3, 4]. Open reduction and internal fixation (ORIF) using plate osteosynthesis for midshaft clavicle fractures is often complicated by the prominence of the surgical implant due to the subcutaneous position of the clavicle. Reoperation rates for symptomatic clavicle plate removal have been reported to be as high as 53%, with female patients being more affected than males [5–7]. We sought to determine to which degree do clinical outcomes differ for plate type following ORIF for midshaft clavicle fractures fixed using superiorly positioned plates by (1) all cause reoperation rate and (2) rate of fracture union.

## Materials and methods

A retrospective chart review was performed using our hospital trauma database for patients treated with open reduction internal fixation for displaced mid-shaft clavicle fractures (OTA/AO type 15-B) between October 2007 and January 2014. Institutional review board approval was granted for the study. Inclusion criteria were patients age greater than 18 years with a minimum of 12 months of clinical follow-up after the index surgery. Surgical indications included acute, mid-shaft clavicle fractures with significant shortening (> 2 cm) or displacement (> 100% of clavicle width), open fractures, impending skin compromise, associated neurological or vascular injury and poly-trauma patients. We excluded any subjects that required operative treatment for a symptomatic nonunion or malunion after failed non-operative treatment, pathological fractures and patients with insufficient radiographs that precluded classification of the fracture pattern. The medical charts and radiographs for the included subjects were reviewed to identify patient demographic information and medical history, mechanism of injury, fracture classification, index surgery characteristics, implant selection and reoperation history. Fractures were classified using the Orthopaedic Trauma Association/Arbeitsgemeinschaft fur Osteosynthesefragen (OTA/AO) criteria [8]. All patients who underwent surgical fixation of their clavicle fracture were treated at a single center. Prophylactic preoperative antibiotics were administrated to the patient before the index surgery was performed [9, 10]. A standard longitudinal incision was utilized and the supraclavicular nerves were identified and preserved whenever possible. All operative cases received a single, superiorly positioned neutralization plate with interfragmentary lag screw compression when possible. Comminuted fracture patterns were treated using a bridge plate technique to span the area of comminution. The choice of surgical plate was left to the operating surgeon and plate types used included 2.7 mm calcaneal reconstruction plates, 2.7 mm reconstruction plates, 3.5 mm pelvic reconstruction plates, 3.5 mm precontoured clavicle plates and 3.5 mm locking compression plates (LCP). All of the implanted plates were manufactured by Synthes, Inc (West Chester, PA). Post-operative management involved sling immobilization for 2 weeks and ongoing clinical and radiographic exams until consolidation of the fracture.

The primary outcome measure was the all cause reoperation rate (including for deep infection, implant failure, non-union and plate prominence). Secondary outcomes included fracture union and documented complications in the study cohort. Data was collected from patient data sheets and patient charts. Data was analyzed using SPSS Version 20 (IBM Corp, Armonk, NY, USA) and descriptive statistics were analyzed. Statistical analysis was performed using Chi square and Fisher Exact test to compare the variables where appropriate. We set our tolerable error for rejecting a true null hypothesis at 0.05. p values < 0.05 were considered statistically significant.

## Results

A total of 102 patients who underwent primary ORIF for a midshaft clavicle fracture were included in the analysis, these were all the patients in our database who meet the inclusion criteria between October 2007 and January 2014. A summary of patient demographics is presented in Table 1. The mean age of patients at the time of surgery was 34.9 ± 12.8 years, with the majority being ≤ 50 years old (83.3%) and male (72.5%). Nineteen patients (18.6%) were smokers (Table 1). The most common mechanism of injury was for bicycle related trauma (31.4%) followed by fall on an out stretched hand (30.4%) (Table 1). Only one of the included fractures was an open fracture. Of the fractures included, 33 (32.4%) were classified as OTA/AO B1 and 69 (67.6%) were B2. Mean time to surgery was 8.6 ± 6.9 days for the cohort. The most commonly used plate was the 3.5 mm reconstruction plate (35.3%) followed by the 2.7 mm calcaneal plate (27.5%) (Table 2). The number of screw holes utilized for each plate construct were eight holes in 41.2% of the cases and 9 holes in 31.4%. In addition a lag screw technique was used in 47.1% of the plating constructs (Table 2).

**Table 1:** Demographic data of clavicle fracture cohort

	Number (%)
Sex	
Male	74 (72.5%)
Female	28 (27.5%)
Age (years)	
≤ 30	47 (46.1%)
31–50	38 (37.7%)
> 50	17 (16.7%)
Hand dominance	
Right	51 (50%)
Left	51 (50%)
Mechanism	
High energy trauma	24 (23.5%)
Low energy trauma	78 (76.5%)
Smoker	
Yes	19 (18.6%)
No	83 (81.4%)
Open fracture	
Yes	1 (1.0%)
No	101 (99.0%)
OTA classification	
B1	33 (32.4%)
B2	69 (67.6%)

**Table 2:** Implants used for open reduction internal fixation of included clavicle fractures

	Number (%)
Plate used	
2.7 mm calcaneal	28 (27.5%)
2.7 mm reconstruction	20 (19.6%)
3.5 mm reconstruction	36 (35.3%)
3.5 mm pre-contoured	8 (7.8%)
3.5 mm locking compression	10 (9.8%)
Number of plate holes utilized	
5	2 (2.0%)
6	9 (8.8%)
7	14 (13.7%)
8	42 (41.2%)
9	32 (31.4%)
10	3 (2.9%)
Lag screw use	
Yes	48 (47.1%)
No	54 (52.9%)

### All cause re-operation rate

Statistically we found that age, sex and smoking were not associated with the re-operation rate. In addition, the OTA/AO fracture classification, time to surgery, type of plate and number of screws used didn’t increase the risk of revision surgery. On the other hand, presence of post-operative pain at 6 weeks was associated with a higher rate of re-operation (*p* value 0.002) as 47.1% of patients with pain at 6 weeks had a secondary surgery, while only 11.8% of patients who were pain free required revision surgery (Table 3). The overall reoperation rate was 17.6%, with plate prominence being the most common cause for re-operation (61.1%) (Table 4).

**Table 3:** Outcome and complications of open reduction internal fixation of clavicle fracture cohort

	Number (%)
Union of fracture	
Yes	99 (97.1%)
No	3 (2.9%)
Cause of hardware failure	
Plate deformation	17 (16.7%)
Screw pullout	8 (7.8%)
Plate breakage	7 (6.9%)
Re-operation	
Yes	18 (17.6%)
No	84 (82.4%)
Reason for reoperation	
Prominence	10 (9.8%)
Plate deformation	3 (2.9%)
Plate breakage	2 (2.0%)
Screw pullout	1 (1.0%)
Infection	1 (1.0%)
Infection	
Superficial	3 (2.9%)
Deep	1 (1.0%)
None	98 (96.1%)
Post-op symptoms at 6 weeks	
Present	17 (16.7%)
None	85 (83.3%)

**Table 4:** Statistical analysis (Chi square) of re-operation rate in clavicle fracture cohort

Patients requiring revision surgery	*n* (%)	d*f*	*p* value
Age			
≤ 30 years (*n* = 47)	8 (17%)	2	0.06
31–50 years (*n* = 38)	10 (26.3%)		
> 50 years (*n* = 17)	0 (0%)		
Sex			
Male (*n* = 74)	12 (16.2%)	1	0.538
Female (*n* = 28)	6 (21.4%)		
Mechanism			
High energy trauma (*n* = 24)	5 (20.8%)	1	0.331
Low energy trauma (*n* = 78)	11 (14.1%)		
Smoking			
Yes (*n* = 19)	5 (26.3%)	1	0.272
No (*n* = 83)	13 (15.7%)		
Open fracture			
Yes (*n* = 1)	0 (0%)	NA^a^	1.0
No (*n* = 101)	18 (17.8%)		
OTA classification			
B1 (*n* = 33)	7 (21.2%)	1	0.514
B2 (*n* = 69)	11 (15.9%)		
Plate used			
2.7 mm calcaneal (*n* = 28)	7 (25%)	4	0.516
2.7 mm reconstruction (*n* = 20)	4 (20%)		
3.5 mm reconstruction (*n* = 36)	6 (16.7%)		
3.5 mm pre-contoured (*n* = 8)	0		
3.5 mm locking compression (*n* = 10)	1 (10%)		
Number of plate holes utilized			
5 (*n* = 2)	1 (50%)	5	0.250
6 (*n* = 9)	2 (22.2%)		
7 (*n* = 14)	2 (14.3%)		
8 (*n* = 42)	4 (11.9%)		
9 (*n* = 32)	9 (28.1%)		
10 (*n* = 3)	0 (0%)		
Post-operative symptoms at 6 weeks			
Pain, (*n* = 17)	8 (47.1%)	3	0.002
None (*n* = 85)	10 (11.8%)		

^a^In these parameters, Fisher’s exact test was used for analysis

### Union rate and documented complications

The union rate was 97.1% for all patients included in the analysis (Table 4). With regards to union rate, there was no association with age, sex, smoking, level of trauma energy, fracture classification, time to surgery or construct characteristics (Table 5). Implant failure was observed in 32 (31.4%) patients; 17 (16.7%) of these patients exhibited plate deformation, 8 (7.8%) had screw pullout and 7 (6.9%) experienced plate breakage (Table 4). In addition, there were no significant associations between documented complications (infection rate and hardware failure) and patient demographics (age and sex), smoking, mechanism of injury and fracture characteristics (Table 6). We found that clavicle fractures fixed with 3.5 mm reconstruction plates were more likely to have hardware failure due to plastic deformation, whereas 2.7 mm constructs where more likely to fail by plate breakage, but these did not reach statistical significance (*p* value = 0.173 and 0.368; respectively) (Table 7). Four patients (3.9%) in our study developed an infection with only 1 case of deep infection that was managed with a revision surgery.

**Table 5:** Statistical analysis (Fisher’s exact test) of union rate in clavicle fracture cohort

Union rate	*n* (%)	*p* value
Age		
≤ 30 years (*n* = 47)	47 (100%)	0.12
31–50 years (*n* = 38)	35 (92.1%)	
> 50 years (*n* = 17)	17 (100%)	
Sex		
Male (*n* = 74)	73 (98.6%)	0.182
Female (*n* = 28)	26 (92.9%)	
Mechanism		
High energy trauma (*n* = 24)	22 (91.7%)	1.0
Low energy trauma (*n* = 78)	77 (98.7)	
Smoking		
Yes (*n* = 19)	19 (100%)	1.0
No (*n* = 83)	80 (96.4%)	
Open fracture		
Yes (*n* = 1)	1 (100%)	1.0
No (*n* = 101)	98 (97.0%)	
OTA classification		
B1 (*n* = 33)	31 (93.9%)	0.244
B2 (*n* = 69)	68 (98.6%)	
Plate used		
2.7 mm calcaneal (*n* = 28)	27 (96.4%)	0.883
2.7 mm reconstruction (*n* = 20)	20 (100%)	
3.5 mm reconstruction (*n* = 36)	34 (94.4%)	
3.5 mm pre-contoured (*n* = 8)	8 (100%)	
3.5 mm locking compression (*n* = 10)	10 (100%)	
Number of plate holes utilized		
5 (*n* = 2)	2 (100%)	0.316
6 (*n* = 9)	8 (88.9%)	
7 (*n* = 14)	13 (92.9%)	
8 (*n* = 42)	41 (97.6%)	
9 (*n* = 32)	32 (100%)	
10 (*n* = 3)	3 (100%)	
Post-operative symptoms at 6 weeks		
Pain, (*n* = 17)	16 (94.1%)	0.425
None (*n* = 85)	83 (97.6%)

**Table 6:** Statistical analysis (Chi square) of documented complications

	Infection rate	d*f*	*p* value	Hardware failure	d*f*	*p* value
Age						
≤ 30 years (*n* = 47)	3 (6.4%)	NA^a^	0.657	13 (27.7%)	2	0.254
31–50 years (*n* = 38)	1 (2.6%)			15 (39.5%)		
> 50 years (*n* = 17)	0			4 (23.5%)		
Sex						
Male (*n* = 74)	4 (5.4%)	NA^a^	0.573	22 (29.7%)	1	0.655
Female (*n* = 28)	0			10 (35.7%)		
Mechanism						
High energy trauma (*n* = 24)	1 (4.2%)	NA^a^	1.0	9 (37.5%)	1	0.743
Low energy trauma (*n* = 78)	3 (3.8%)			23 (29.5%)		
Smoking						
Yes (*n* = 19)	1 (5.3%)	NA^a^	0.568	9 (47.4%)	1	0.173
No (*n* = 83)	3 (3.6%)			23 (27.7%)		
Open fracture						
Yes (*n* = 1)	0	NA^a^	1.0	1 (100%)	NA^a^	0.324
No (*n* = 101)	4			31 (30.7%)		
OTA classification						
B1 (*n* = 33)	0	NA^a^	0.302	9 (27.3%)	1	0.448
B2 (*n* = 69)	4 (58.0%)			23 (33.3%)		

^a^In these parameters, Fisher’s exact test was used for analysis

**Table 7:** Statistical analysis (Fisher’s exact test) of hardware failure by type of plate utilized

Hardware failure	2.7 mm calcaneal plate (*n* = 28)	2.7 mm reconstruction plate (*n* = 20)	3.5 mm reconstruction plate (*n* = 36)	3.5 mm pre-contoured plate (*n* = 8)	3.5 mm locking compression plate (*n* = 10)	*p* value
Plastic deformation	3 (10.7%)	2 (10%)	10 (27.8%)	0	2 (20%)	0.222
Plate breakage	4 (14.3%)	1 (5%)	1 (2.8%)	0	1 (10%)	0.397
Screw pullout	0	3 (15%)	5 (13.9)	0	0	0.128

## Discussion

This study examines the effect of patient demographics, mechanism of injury and plating construct characteristics on the reoperation, union and complication rates after open reduction and internal fixation of mid-shaft clavicle fractures. Our results demonstrated an all cause reoperation rate of 17.6% with plate prominence being the most common cause of re-operation (61.1%). This is similar to the results published in a recent study by Leroux et al. [5], examining the incidence and associated risk factors for reoperations after ORIF of midshaft clavicle fractures. The authors found that out of 1350 patients, almost one in four (24.6%) underwent a subsequent clavicle operation within 2 years of the index surgery with the most common reoperation procedure being performed for isolated implant removal (18.8%) [5]. In addition, results from the Canadian Orthopaedic Trauma Society (COTS) trial reported that 8% of clavicle fractures treated with open reduction and internal fixation would require an implant removal procedure within 1 year of the initial procedure [4].

The need for plate removal is often due to plate prominence and poor cosmesis due to the subcutaneous position of the clavicle [6]. We found no statistically significant difference between 2.7 and 3.5 mm plates on the rate or reoperation nor was there any difference between 2.7 and 3.5 mm plates on the rate of post-operative pain or numbness 6 weeks post-surgery. Our results are similar to those reported by Galdi et al. [11], who examined whether using a lower profile 2.7 mm reconstruction plate would lead to better clinical outcomes and lower removal rates when compared to a 3.5 mm reconstruction plate for ORIF of OTA/AO type B clavicle fractures. The authors reported that using the thinner 2.7 mm plates provided for higher rates of cosmetic acceptability, but did not demonstrate a statistically significant difference with regards to reoperation rates for plate removal when compared to 3.5 mm plates [11].

Our reported overall rate of nonunion of 2.9% is comparable with previously published research. Zlowodzki et al. showed a 2.5% nonunion rate in a systematic review of 635 clavicle fractures treated operatively [12]. Also in a recent meta-analysis, McKee et al. identified a nonunion rate of 1.4% of clavicle fracture ORIFs [13]. We did not find an association between nonunion and age, sex, smoking, level of trauma energy, fracture classification, time to surgery or construct characteristics. Leroux et al. found that both female gender and a high comorbidity score (Collapsed Aggregate Diagnosis Group) increased the odds of nonunion in their large cohort [5].

In addition, clavicle fractures fixed with 3.5 mm reconstruction plates were more likely to exhibit plastic deformation, whereas 2.7 mm plating constructs utilizing reconstruction plates where more likely to fail by plate breakage. These results are in keeping with previous clinical studies that have demonstrated the susceptibility of 3.5 and 2.7 mm reconstruction plates to undergo plastic deformation when used for open reduction internal fixation of midshaft clavicle fractures [14–17]. Moreover, our infection rate was 3.9%, of which only 1 case required a revision surgery for deep infection. This rate was comparable to available literature where the rate for postoperative infection ranges from 2.6 to 10% [5, 7].

The major limitation of our study is the retrospective design. Despite reporting on a large series of operatively treated clavicle fractures, we were unable to detect a significant difference between plate types with regards to union rates, need for surgical reoperation, incidence of plate removal and the rate of implant failure. Future studies with a larger sample size may detect these differences if present.

This retrospective study demonstrated an overall reoperation rate of 17.6% for midshaft clavicle fractures treated with open reduction internal fixation utilizing both 2.7 and 3.5 mm plates. Presence of symptoms (pain and numbness) at the surgical site was associated with an increased risk of re-operation. Although union rate and post-operative complications had no association with the type of plate used, future studies with a larger sample size are required to further examine these outcomes.
